# Supplementary material for: Image-based phenotyping of disaggregated cells using deep learning
Source: Commun Biol. 2020 Nov 13;3:674. doi: 10.1038/s42003-020-01399-x (PMC7666170; doi:10.1038/s42003-020-01399-x)
Supplement: Supplementary file 1 — Supplementary Information [file 42003_2020_1399_MOESM1_ESM.pdf]

# Image-based Cell Phenotyping Using Deep Learning

Samuel Berryman[1,2], Kerryn Matthews[1,2], Jeong Hyun Lee[1,2], Simon P. Duffy[1,2,3], and Hongshen Ma[1,2,4,5*]

**Affiliations:**
[1]Department of Mechanical Engineering, University of British Columbia
[2]Centre for Blood Research, University of British Columbia
[3]British Columbia Institute of Technology
[4]School of Biomedical Engineering, University of British Columbia
[5]Department of Urologic Sciences, University of British Columbia

* Correspondence should be addressed to Hongshen Ma (hongma@mech.ubc.ca)

## Supplemental Images

The following contains sample fluorescent images that were both accepted and rejected by the pre-processing pipeline. The brightfield channel was excluded as only three channels are available to display in an image and overlaying makes the fluorescent channels more difficult to analyze.

Note that each of the channels was scaled so that the minimum value was zero and the maximum value was 255.

## Accepted image patches:

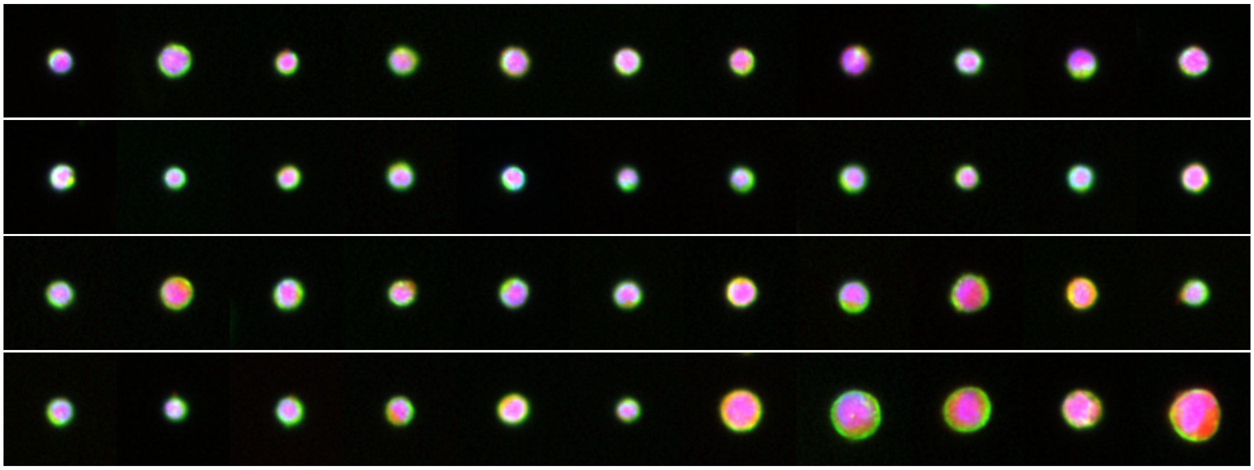

**Figure S1 | Sample HCT Cell Images**. Sample segmented fluorescent microscopy images of HCT116 cells. Fluorescent channels are Hoechst (Blue), Sir-Actin (Red) and Calcein (Green). Each channel is shown here normalized between 0 and 255.

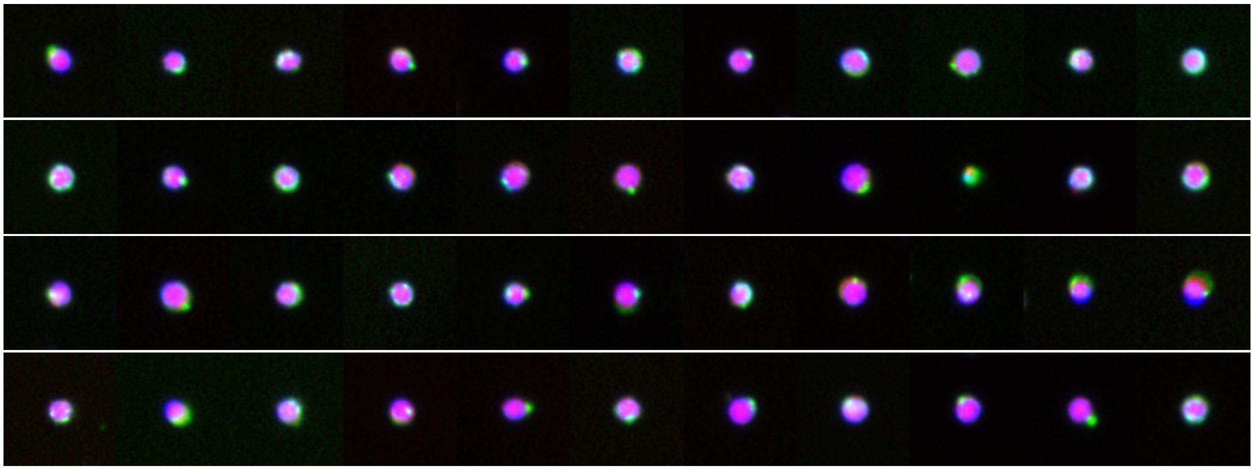

**Figure S2 | Sample HL60 Cell Images**. Sample segmented fluorescent microscopy images of HL60 cells. Fluorescent channels are Hoechst (Blue), Sir-Actin (Red) and Calcein (Green). Each channel is shown here normalized between 0 and 255.

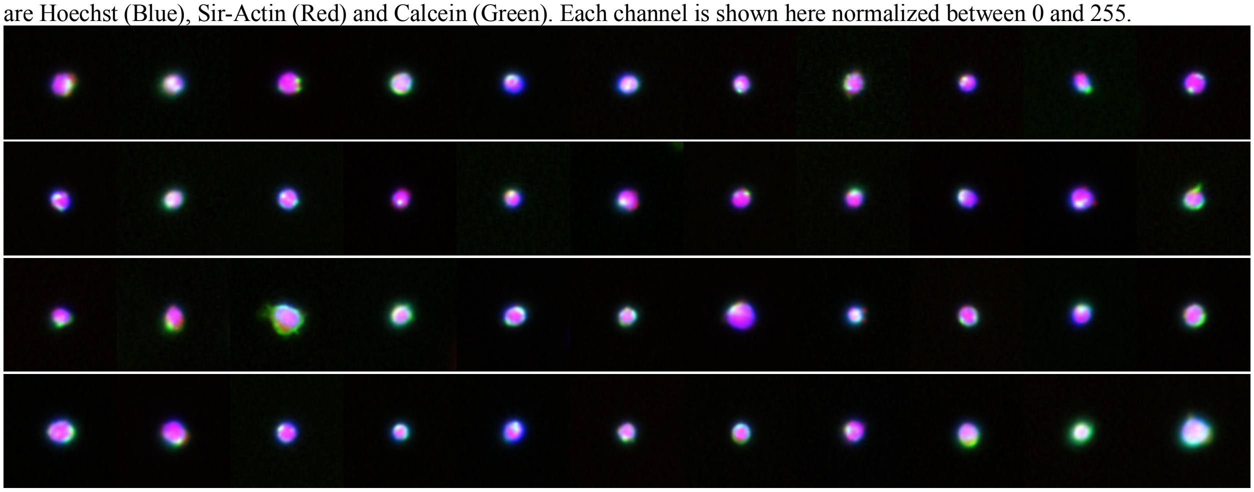

**Figure S3 | Sample Jurkat Cell Images**. Sample segmented fluorescent microscopy images of Jurkat cells. Fluorescent channels are Hoechst (Blue), Sir-Actin (Red) and Calcein (Green). Each channel is shown here normalized between 0 and 255.

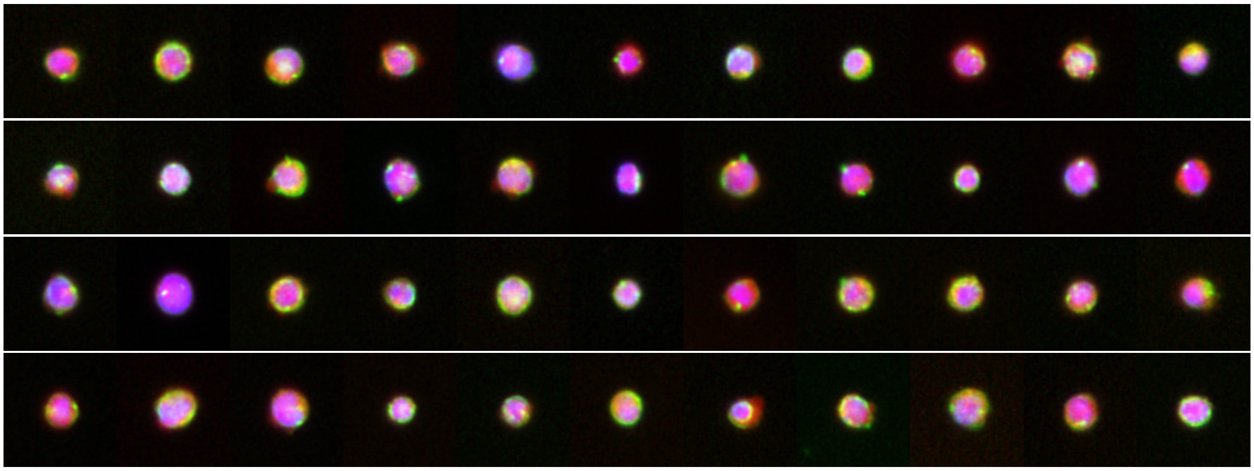

**Figure S4 | Sample LNCaP Cell Images**. Sample segmented fluorescent microscopy images of LNCaP cells. Fluorescent channels are Hoechst (Blue), Sir-Actin (Red) and Calcein (Green). Each channel is shown here normalized between 0 and 255.

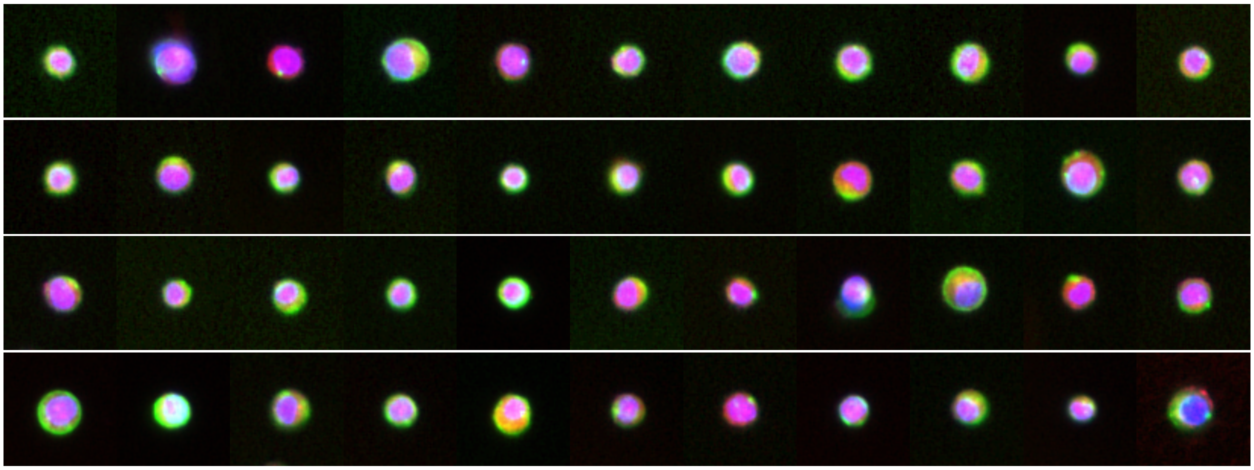

**Figure S5 | Sample MCF7 Cell Images**. Sample segmented fluorescent microscopy images of MCF7 cells. Fluorescent channels are Hoechst (Blue), Sir-Actin (Red) and Calcein (Green). Each channel is shown here normalized between 0 and 255.

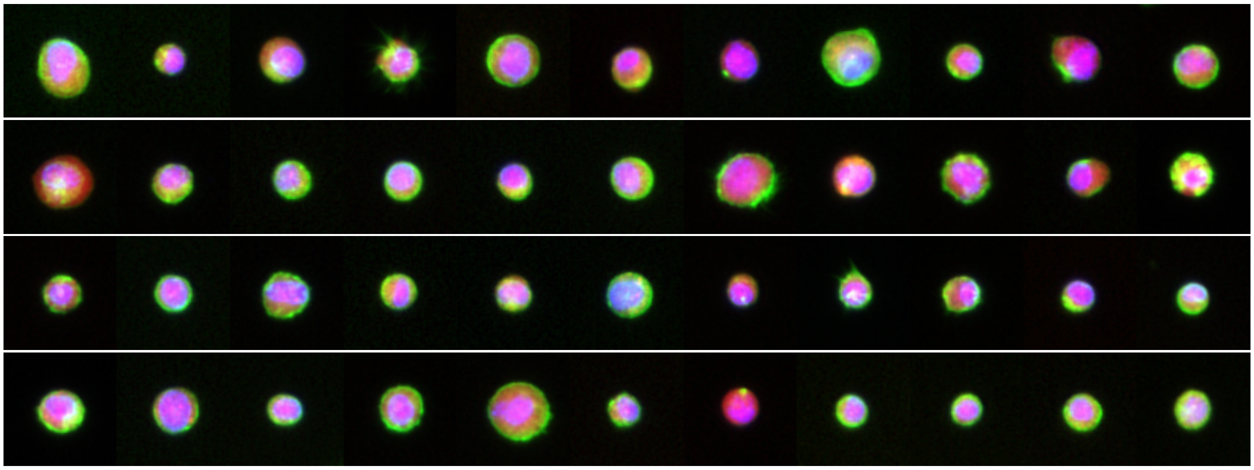

**Figure S6 | Sample PC3 Cell Images**. Sample segmented fluorescent microscopy images of PC3 cells. Fluorescent channels are Hoechst (Blue), Sir-Actin (Red) and Calcein (Green). Each channel is shown here normalized between 0 and 255.

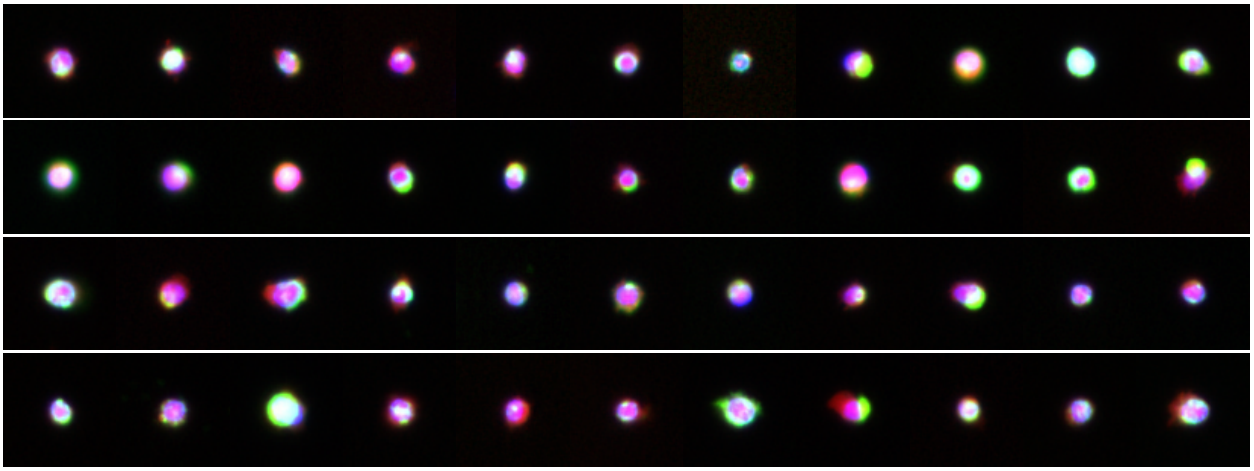

**Figure S7 | Sample THP-1 Cell Images**. Sample segmented fluorescent microscopy images of THP-1 cells. Fluorescent channels are Hoechst (Blue), Sir-Actin (Red) and Calcein (Green). Each channel is shown here normalized between 0 and 255.

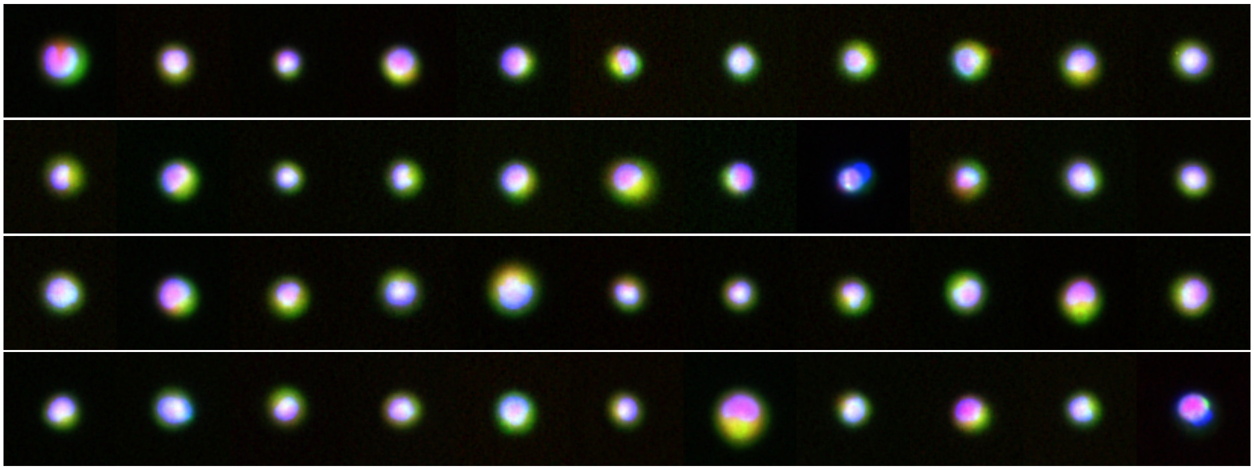

**Figure S8 | Sample U2OS Cell Images**. Sample segmented fluorescent microscopy images of U2OS cells. Fluorescent channels are Hoechst (Blue), Sir-Actin (Red) and Calcein (Green). Each channel is shown here normalized between 0 and 255.

## Rejected Image Patches:

The pre-processing pipeline was designed to reject samples that didn't contain a single cell.

### The following image patches samples were rejected for having to many cells present in the image patch:

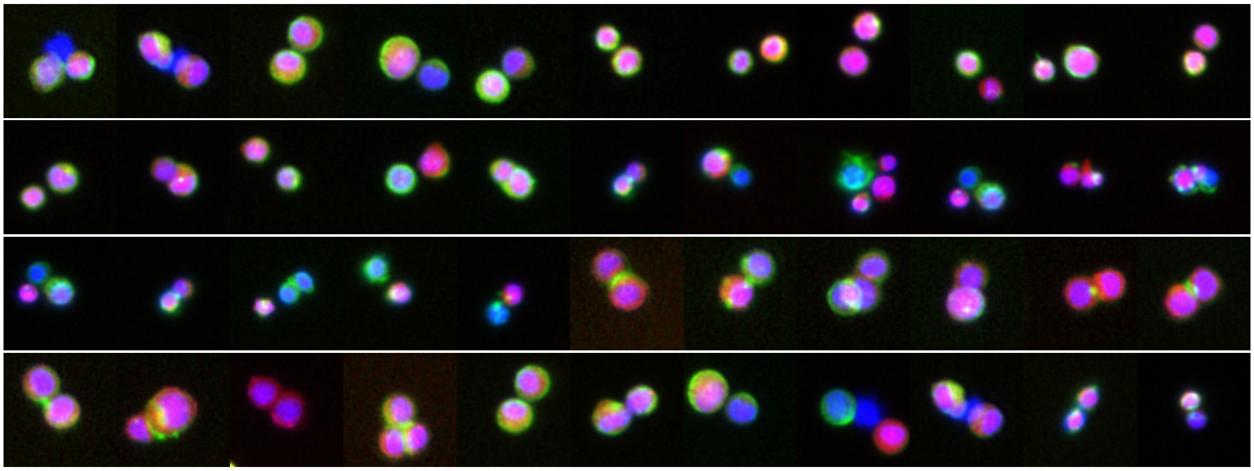

**Figure S9 | Multiple Cell Rejection**. Sample segmented fluorescent microscopy images rejected due to containing multiple cells. Fluorescent channels are Hoechst (Blue), Sir-Actin (Red) and Calcein (Green). Each channel is shown here normalized between 0 and 255.

### The following image patch samples were rejected for having an object on the image border:

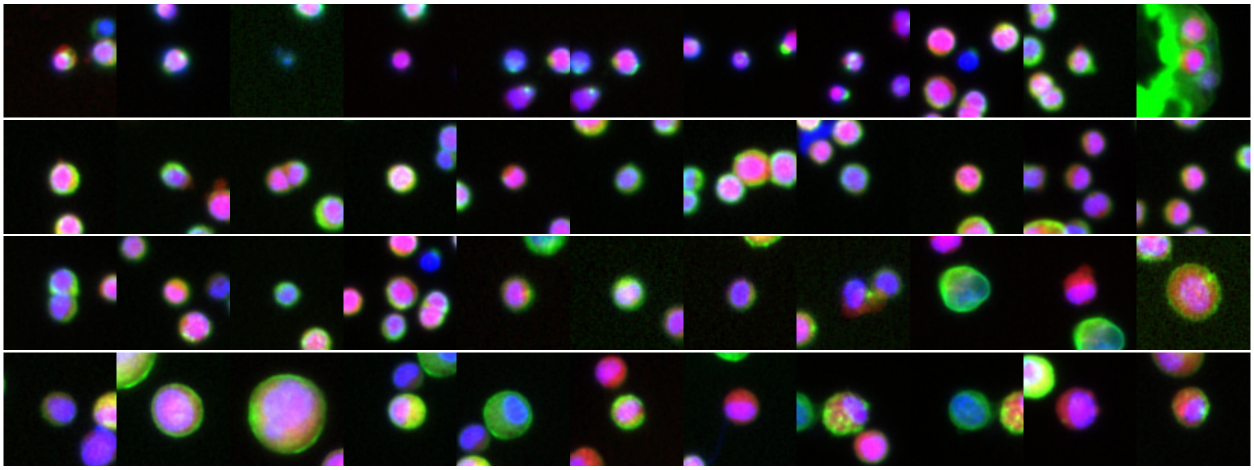

**Figure S10 | Image Boundary Rejection**. Sample segmented fluorescent microscopy images rejected due to having objects lying on the image boundary. Fluorescent channels are Hoechst (Blue), Sir-Actin (Red) and Calcein (Green). Each channel is shown here normalized between 0 and 255.

**The following image patches samples were rejected for not having enough Calcium-AM present (were not viable) Note that the image is extremely noisy due to the lack of Calcium-AM stain in the image:**

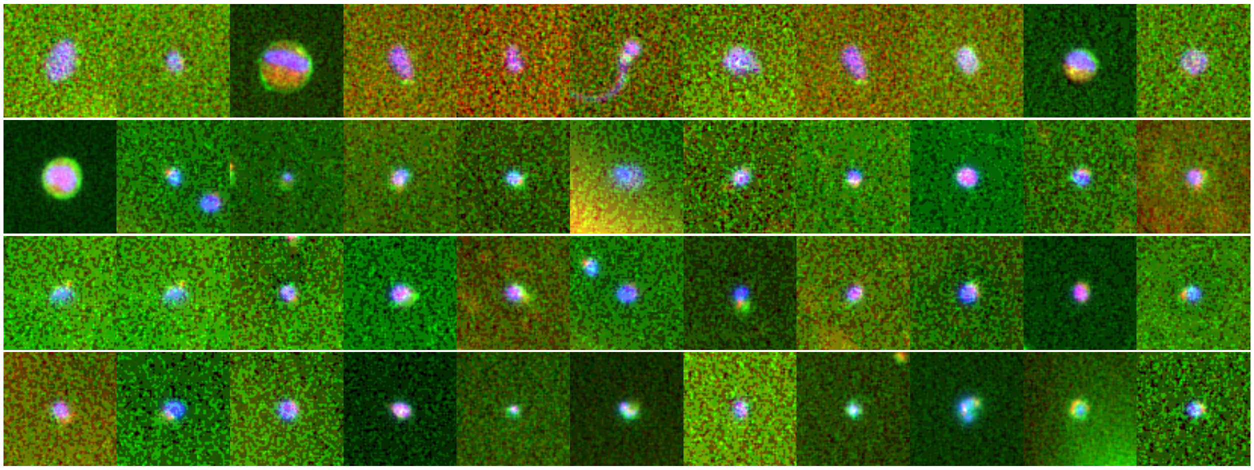

**Figure S11 | Viability Rejection**. Sample segmented fluorescent microscopy images rejected due to cells being determined to be not-viable. Fluorescent channels are Hoechst (Blue), Sir-Actin (Red) and Calcein (Green). Each channel is shown here normalized between 0 and 255. The images have an extremely high level of noise due to the lack of Calcein-AM stain in the images.

| | HCT | HL60 | Jurkat | LNCaP | MCF7 | PC3 | THP-1 | U2OS |
|---|---|---|---|---|---|---|---|---|
| HCT | 3244 | 4 | 7 | 16 | 3 | 21 | 0 | 11 |
| HL60 | 7 | 1224 | 21 | 7 | 10 | 12 | 11 | 21 |
| Jurkat | 1 | 6 | 3936 | 11 | 3 | 3 | 17 | 3 |
| LNCaP | 5 | 0 | 93 | 1061 | 35 | 9 | 4 | 9 |
| MCF7 | 1 | 1 | 1 | 19 | 1650 | 23 | 0 | 7 |
| PC3 | 0 | 0 | 2 | 4 | 30 | 1871 | 0 | 1 |
| THP-1 | 1 | 24 | 1 | 2 | 1 | 0 | 1276 | 66 |
| U2OS | 5 | 3 | 3 | 9 | 4 | 9 | 17 | 1019 |

**Figure S12 | Non-normalized Confusion Matrix,** Confusion matrix constructed on the entire testing dataset. Diagonal values correspond to number of correct classifications. Graphs in each position are the cumulative frequencies of classification probabilities for the corresponding classes.
